# Genetic variation of naturally growing olive trees in Israel: from abandoned groves to feral and wild?

**DOI:** 10.1186/s12870-016-0947-5

**Published:** 2016-12-13

**Authors:** Oz Barazani, Alexandra Keren-Keiserman, Erik Westberg, Nir Hanin, Arnon Dag, Giora Ben-Ari, Ori Fragman-Sapir, Yizhar Tugendhaft, Zohar Kerem, Joachim W. Kadereit

**Affiliations:** 1 Institute of Plant Sciences, the Israel Plant Gene Bank, Agricultural Research Organization, Rishon LeZion, 75359 Israel; 2 Herbarium, the National Natural History Collections, the Hebrew University of Jerusalem, Jerusalem, 91904 Israel; 3 Institut für Spezielle Botanik und Botanischer Garten, Johannes Gutenberg-Universität Mainz, D-55099 Mainz, Germany; 4 Institute of Plant Sciences, Department of Fruit Tree Sciences, Agricultural Research Organization, Gilat Research Center, Gilat, 85280 Israel; 5 Institute of Plant Sciences, Department of Fruit Trees Sciences, Agricultural Research Organization, Rishon LeZion, 75359 Israel; 6 Jerusalem Botanical Gardens, the Hebrew University, Giv’at Ram, Jerusalem, 9021904 Israel; 7 Institute of Biochemistry, Food Science and Nutrition, Robert H. Smith Faculty of Agriculture, Food and Environment, the Hebrew University of Jerusalem, Rehovot, 76100 Israel

**Keywords:** Crop domestication, Cultivated old olive trees, Gene flow, Grafting, Historical agriculture, Oleaster, var. *sylvestris*

## Abstract

**Background:**

Naturally growing populations of olive trees are found in the Mediterranean garrigue and maquis in Israel. Here, we used the Simple Sequence Repeat (SSR) genetic marker technique to investigate whether these represent wild var. *sylvestris*. Leaf samples were collected from a total of 205 trees at six sites of naturally growing olive populations in Israel. The genetic analysis included a multi-locus lineage (MLL) analysis, Rousset’s genetic distances, Fst values, private alleles, other diversity values and a Structure analysis. The analyses also included scions and suckers of old cultivated olive trees, for which the dominance of one clone in scions (MLL1) and a second in suckers (MLL7) had been shown earlier.

**Results:**

The majority of trees from a Judean Mts. population and from one population from the Galilee showed close genetic similarity to scions of old cultivated trees. Different from that, site-specific and a high number of single occurrence MLLs were found in four olive populations from the Galilee and Carmel which also were genetically more distant from old cultivated trees, had relatively high genetic diversity values and higher numbers of private alleles. Whereas in two of these populations MLL7 (and partly MLL1) were found in low frequency, the two other populations did not contain these MLLs and were very similar in their genetic structure to suckers of old cultivated olive trees that originated from sexual reproduction.

**Conclusions:**

The genetic distinctness from old cultivated olive trees, particularly of one population from Galilee and one from Carmel, suggests that trees at these sites might represent wild var. *sylvestris*. The similarity in genetic structure of these two populations with the suckers of old cultivated trees implies that wild trees were used as rootstocks. Alternatively, trees at these two sites may be remnants of old cultivated trees in which the scion-derived trunk died and was replaced by suckers. However, considering landscape and topographic environment at the two sites this second interpretation is less likely.

**Electronic supplementary material:**

The online version of this article (doi:10.1186/s12870-016-0947-5) contains supplementary material, which is available to authorized users.

## Background

The domestication of crop species started 13,000 to 10,000 years before present by gradual selection of desirable traits and of adaptations to agricultural environments [1]. Such artificial selection of individual plants with desirable traits, e.g., high yield, large fruits, loss of shattering seeds, etc., had an artificial selection effect which resulted in genetic differences between crops and their wild ancestors, both in coding and neutral regions of the genome. However, the long co-existence of crops alongside their wild relatives provided opportunities for hybridization, leading to gene flow between the diverging gene pools. Gene flow between cultivated plants and their wild ancestors has been demonstrated in woody species cultivated for their edible fruits such as almonds (*Prunus dulcis* and *P. orientalis*) [2], grapes (*Vitis vinifera* subsp. *vinifera* and *V. vinifera* subsp. *sylvestris*) [3, 4] and apples (*Malus domestica* and *M. sylvestris*) [5]. In addition to gene flow, dispersal of seeds from cultivated trees into natural surroundings can result in feral populations of natural aspect [6], as shown for several plants introduced to Australia, including *Olea europaea* [7, 8]. Both these processes can result in substantial difficulties when trying to identify populations as truly wild.

It is generally accepted that the cultivated olive *Olea europaea* subsp. *europaea* var. *europaea* originated from wild var. *sylvestris* (Mill) Lehr by artificial selection from wild populations [9]. Recently, analysis of plastid DNA diversity among 1,263 supposedly wild olive trees from 108 localities across the Mediterranean area and 534 cultivars suggested that the north Levant (i.e., the area close to the Syrian/Turkish border) was the primary domestication centre of olives [10]. However, one of the earliest indications of the use of olives and possibly also of its cultivation was found in the southeastern Mediterranean area (i.e., in the area of modern Israel) and dated to 6,500 B.C. [11].

Wild var. *sylvestris*, often called ‘oleaster’, resembles cultivated olives except for its shrubby growth and smaller leaves and fruits [12]. These characters, however, are highly variable and do not allow reliable distinction between the wild and cultivated varieties. Thus, the identification of olives growing in natural surroundings in the southeast Mediterranean area as var. *sylvestris* is often questionable [13]. However, using an ecological niche model based on current climatic parameters, Besnard et al. [10] could identify the natural distribution range of var. *sylvestris* and could show that current conditions are suitable for its presence in the southwest Levant, i.e., modern Israel.

Studies employing different molecular marker techniques to investigate the relationship between cultivated and wild olives and to map the distribution of wild olives in the Mediterranean area have been conducted before, e.g. [14–22]. In several cases, genetic similarity between trees growing in natural surroundings and cultivated olives was interpreted as evidence for the feral nature (i.e., descended from cultivated trees) of the former [14, 15]. However, the studies by Baldoni et al. [14] and Belaj et al. [15] also revealed the existence of genetically distinct populations in Italy and Spain, respectively, which were interpreted as evidence for the continued existence of isolated populations of wild var. *sylvestris* in the Mediterranean area. Supporting this hypothesis, other studies using DNA [22–24] and allozyme [19] variation differentiated between cultivated and wild forms of olives. More recently, a comprehensive Bayesian analysis of microsatellite variation that included cultivated and supposedly wild trees from around the Mediterranean Basin showed that wild trees from the southeastern Mediterranean region were genetically closely similar to Spanish cultivars [25]. The study by Diez et al. [25] as well as others [15, 26] thus suggest that the identification of naturally growing populations of olives as var. *sylvestris* requires caution in view of the possibility of gene flow between cultivated and wild populations.

In Israel, naturally growing populations of olive trees can be found in the Mediterranean maquis and garrigues of the Carmel and western Galilee mountain ranges. Considering that it is likely that olives have been cultivated continuously in the area for at least 6,000 years [11, 20, 27–30], and that olive groves occupy large parts of the rural landscape, the continued existence of populations of var. *sylvestris* in the region perhaps is not likely and needs to be studied. Several studies included samples of naturally growing olive trees from the southeastern Mediterranean to infer the distribution and genetic diversity among population of ‘oleaster’ around the Mediterranean [17, 19, 25]. Higher genetic diversity was found in populations of naturally growing olive trees in the west Mediterranean than in the East Mediterranean area, suggesting the existence of genuine var. *sylvestris* in the west Mediterranean [17] but questioning the status of naturally growing olive trees in the southeastern Mediterranean. The genetic variation of populations of var. *sylvestris* potentially could have enormous importance in breeding programs aiming at the introduction of wild alleles conferring valuable traits that were lost during the domestication process [31]. On this background, knowledge of the status of naturally growing populations of olives is of high importance for developing conservation programs for this valuable germplasm. Conservation efforts should also address the risks of hybridization and introgression from domesticated crops into populations of their wild relatives [32], as recently shown for fruit trees [2, 5].

To determine the identity of naturally growing olive populations in Israel as wild var. *sylvestris*, feral (var. *europaea*) or perhaps as abandoned groves, we used SSR markers for the analysis of six naturally growing olive populations sampled at close to far distances from extant cultivated groves. In a previous study we already used a multi-locus lineage (MLL) analysis with the same SSR markers to infer cultivar identity in the same region [33]. We could show the dominance of one clone in scions, and that another clone is frequent in rootstocks of grafted trees. We used these earlier results to assess genetic similarity between supposedly wild populations and local old cultivated olive trees. More specifically, we hypothesize that genuinely wild populations (i.e., var. *sylvestris*), if such exist, will be genetically different from cultivated olives and feral populations. On this background, a population of naturally growing olives from outside the hypothetical natural distribution range of wild olives in the region [9] was included as potential reference as a feral population.

## Results

### Multi-locus lineage analysis

**Fig. 1:**
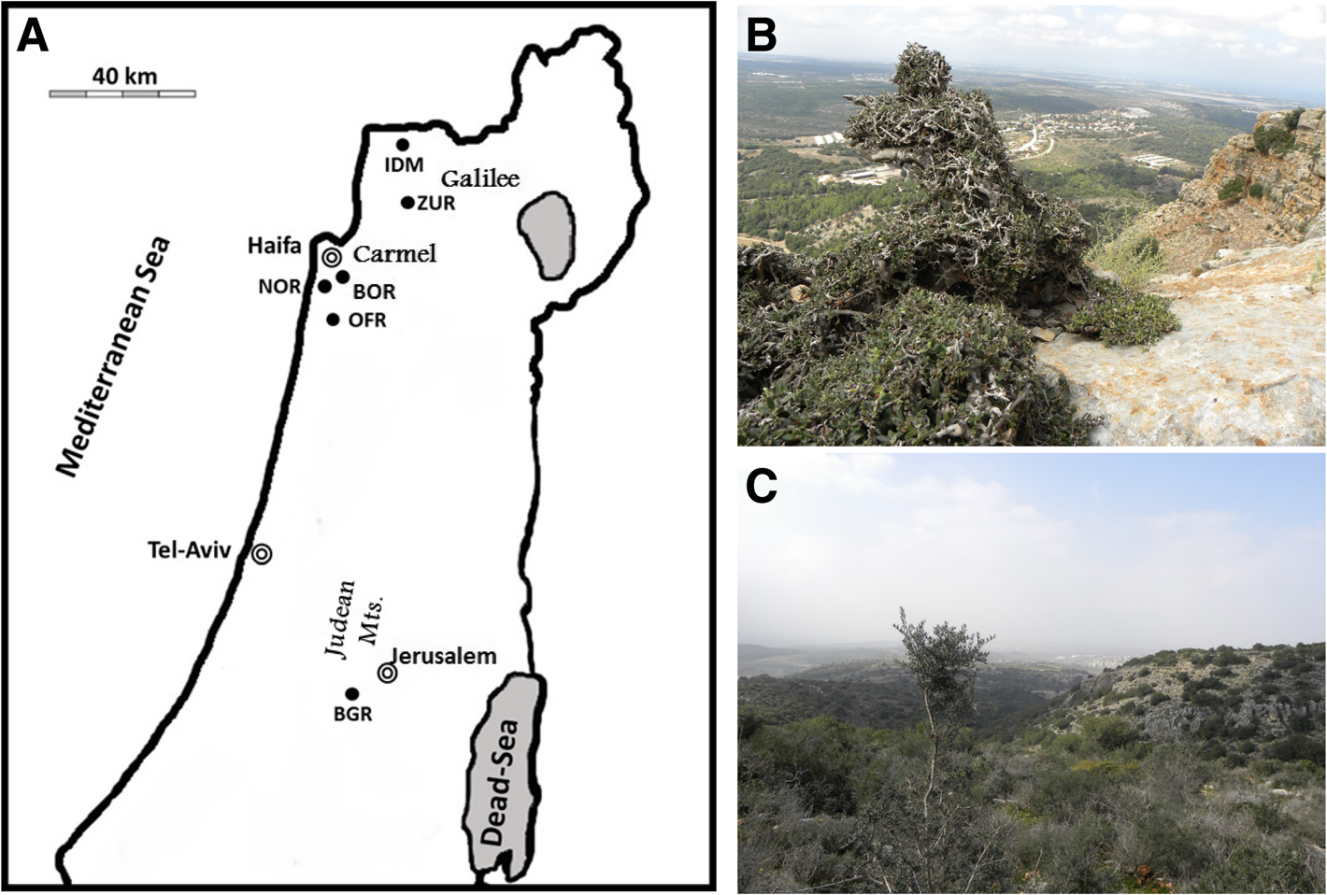
Location of the six naturally growing olive populations sampled (**a**); naturally growing olive trees in the Galilee at Idmit, where trees are exposed to strong herbivore pressure (**b**) and in a typical garrigue formation at Zurit (**c**)

The genetic analysis included 205 naturally growing olive trees sampled in six populations in Israel (Table 1; Fig. 1). Using 15 SSR markers, the number of alleles per locus in the total of 205 trees ranged from four to 32 (Additional file 1: Table S1). Raw microsatellite data for the 15 markers is available in the Additional file 2: Table S2. Analysis of multi-locus genotypes (MLGs) and grouping of MLGs into multi-locus lineages (MLLs) reduced the probability of mistakes resulting from SSR genotyping errors, thus permitting the comparison of naturally growing populations with grafted old olive trees [30]. The multi-locus lineage analysis (Table 2) showed that 10 trees each of the naturally growing and cultivated trees at BGR belonged to the most commonly cultivated regional clone MLL1 [33]. One of the remaining nine trees belonged to MLL7 and the remaining eight trees were assigned to site-specific single occurrence MLLs of which seven were found in naturally growing trees. In ZUR, only three distinct MLLs were found, with 20 trees assigned to the cultivated MLL1, one to MLL7, and only two to a site-specific MLL. In contrast, trees sampled in IDM (Galilee) and the Carmel populations NOR, BOR and OFR generally belonged to site specific MLLs. Whereas both BOR and OFR contained a small number of trees with MLL7, and the two sampled cultivated trees in OFR belonged to MLL1, neither MLL1 nor MLL7 were found in IDM and NOR. Other than these two common MLLs, no MLLs were shared between populations (Table 2 and Additional file 3: Table S3).

**Table 1:** Naturally growing olive populations used in this study and their geographical distribution (c.f. Fig. 1a) within (Galilee and Carmel) and outside (Judean Mts.) the hypothetical natural distribution range of var. *sylvestris* [9]

Population		Sample size	Coordinates		
				Longitude	Latitude
Galilee	Idmit	25	IDM	E 35° 11′ 41.86″	N 33° 04′ 35.54″
	Zurit	23	ZUR	E 35° 13′ 19.33″	N 32° 55′ 44.48″
Carmel	Nachal Oren	35	NOR	E 34° 58′ 39.31″	N 32° 42′ 48.85″
	Beit Oren	54	BOR	E 35° 01′ 09.48″	N 32° 43′ 58.45″
	Ofer	39	OFR	E 34° 59′ 41.06″	N 32° 37′ 33.79″
Judean Mts.	Bar Giora	29	BGR	E 35° 04′ 22.58″	N 31° 44′ 53.98″

**Table 2:** Number of olive trees assigned to different multi-locus lineages (MLL) using 15 SSR markers

MLL	IDM	ZUR	NOR	BOR	OFR	BGR		Suckers	Scions
						C	Wild		
1	·	20	·	·	2	10	10	65	260
2	·	·	·	·	·	·	·	1	1
3	·	·	·	·	·	·	·	3	1
4	·	·	·	·	·	·	·	3	1
5	·	·	·	·	·	·	·	2	1
6	·	·	·	·	·	·	·	1	1
7	·	1	·	2	2	·	1	69	11
8	·	·	·	·	·	·	·	1	1
9	·	·	·	·	·	·	·	2	1
**10**	·	·	·	·	·	·	·	·	**1**
**11**	·	·	·	·	·	·	·	·	**2**
12	·	·	·	·	·	·	·	1	1
13	·	·	·	·	·	·	·	1	1
14	·	·	·	·	·	·	·	1	1
15	·	·	·	·	·	·	·	1	1
16	·	·	·	·	·	·	·	1	1
17	·	·	·	·	·	·	·	1	1
18	·	·	·	·	·	·	·	1	1
**24**	·	·	·	·	·	·	·	**2**	·
**66**	·	·	·	·	·	·	·	**2**	·
**144**	**6**	·	·	·	·	·	·	·	·
**146**	**4**	·	·	·	·	·	·	·	·
**147**	**2**	·	·	·	·	·	·	·	·
**149**	**2**	·	·	·	·	·	·	·	·
**151**	**3**	·	·	·	·	·	·	·	·
**170**	·	·	·	·	**2**	·	·	·	·
**175**	·	·	·	·	**4**	·	·	·	·
**182**	·	·	·	·	**2**	·	·	·	·
**193**	·	·	**2**	·	·	·	·	·	·
**217**	·	·	**4**	·	·	·	·	·	·
221	·	·	·	**3**	·	·	·	·	·
233	·	·	·	**4**	·	·	·	·	·
249	·	·	·	**2**	·	·	·	·	·
250	·	·	·	**3**	·	·	·	·	·
251	·	·	·	**2**	·	·	·	·	·
**269**	·	**2**	·	·	·	·	·	·	·
SO	**8**	·	**29**	**38**	**27**	**1**	**7**	**123**	**1**
Total	13	3	31	44	32	2	9	141	18

The number of trees assigned to each MLL and the total number of MLLs found in each population are given. For comparison, MLLs of suckers and scions of cultivated old olive trees are indicated. Site-specific and single occurrence (SO) MLLs are indicated in bold; MLL1 and 7 represent the most common MLLs found in scions and suckers of old cultivated trees, respectively [33]. MLLs in the BGR population represent the supposedly cultivated (C) and naturally growing (wild) trees

In comparison, samples of suckers (collected at the base of tree trunks) and scions, from presumably grafted trees, belonged to 141 and 18 MLLs, respectively (Table 2; [33]). Of the total of 269 MLLs (Additional file 3: Table S3), 16 were shared by scions and suckers, two were scion specific (MLL10 and 11) and 125 were specific to suckers, the majority of them as single occurrence MLLs (Table 2 and Additional file 3: Table S3).

### Genetic diversity estimates

The genetic diversity values (Table 3) showed that values of allelic richness and mean number of private alleles per locus found in IDM and NOR were higher than those found in the other populations. The average number of private alleles per locus in OFR (0.49) was the lowest among the six populations and in comparison to scions (0.53) and suckers (0.57). No noticeable differences among populations and cultivated trees were found in observed and unbiased expected heterozygosity values (Table 3).

**Table 3:** Observed (Ho) and unbiased expected (uHe) heterozygosity, allelic richness (Ar) and mean number of private alleles per locus (Pr. Al) in the populations analyzed

	Ho	uHe	Ar	Pr.Al
IDM	0.77	0.80	7.28	1.00
ZUR	0.87	0.72	-	-
NOR	0.76	0.79	7.39	0.90
BOR	0.79	0.77	6.56	0.79
OFR	0.74	0.72	5.75	0.49
BGR	0.75	0.72	5.69	0.61
Suckers	0.74	0.74	6.20	0.57
Scions	0.77	0.72	5.59	0.53

Diversity values were calculated for one individual sample per MLL (Table 2); data for old cultivated olive trees, sucker and scions were extracted from Barazani et al. (2014) [33]

**Fig. 2:**
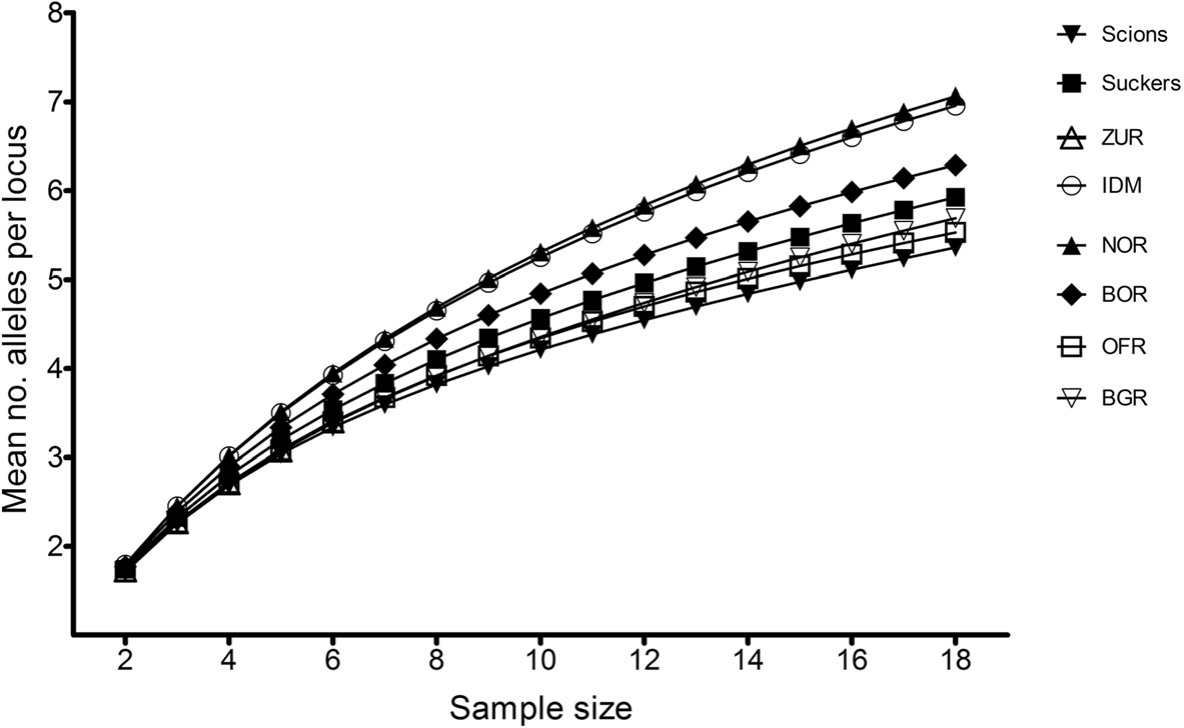
Mean number of alleles per locus as a function of sample size of the populations analyzed and of suckers and scions of old cultivated trees

Further analysis of private allelic richness indicated that IDM and NOR had the highest number of private alleles per locus (Fig. 2) when corrected for sample size using ADZE [34]. The number of private alleles in 112 different combinations of populations is presented in the Additional file 4: Figure S1. The highest number of private alleles shared by two populations was found in the combination of IDM and NOR (Additional file 4: Figure S1A). In the combination of three populations, the highest number of private alleles was found in IDM together with BOR and NOR, and in the combination of four populations in IDM, BOR, NOR and OFR. The combination of these four populations with suckers yielded the highest number of private alleles per locus (combination of five populations; Additional file 4: Figure S1D). Any combination of suckers from IDM, BOR, NOR and OFR with scion MLLs resulted in smaller numbers of private alleles per locus (Additional file 4: Figure S1A-D). In combinations of two populations with suckers, the number of private alleles per locus was substantially higher in IDM and NOR with suckers than in BOR and OFR with suckers (Additional file 4: Figure S1B).

### Genetic differentiation among wild growing populations and cultivated olives

The pairwise Fst analysis (Table 4) revealed that BGR and OFR are most similar to each other (Fst = 0.016), while the highest genetic differentiation was found between populations ZUR and IDM (Fst = 0.061) and between ZUR and NOR and BOR (Fst = 0.052 and 0.051, respectively). BOR and NOR were very similar to IDM (Table 4).

**Table 4:** Pairwise Fst values between naturally growing olive populations

	IDM	ZUR	NOR	BOR	OFR	BGR
IDM	0.000					
ZUR	0.061	0.000				
NOR	0.019	0.052	0.000			
BOR	0.026	0.051	0.025	0.000		
OFR	0.033	0.037	0.023	0.034	0.000	
BGR	0.039	0.039	0.032	0.042	0.016	0.000

**Fig. 3:**
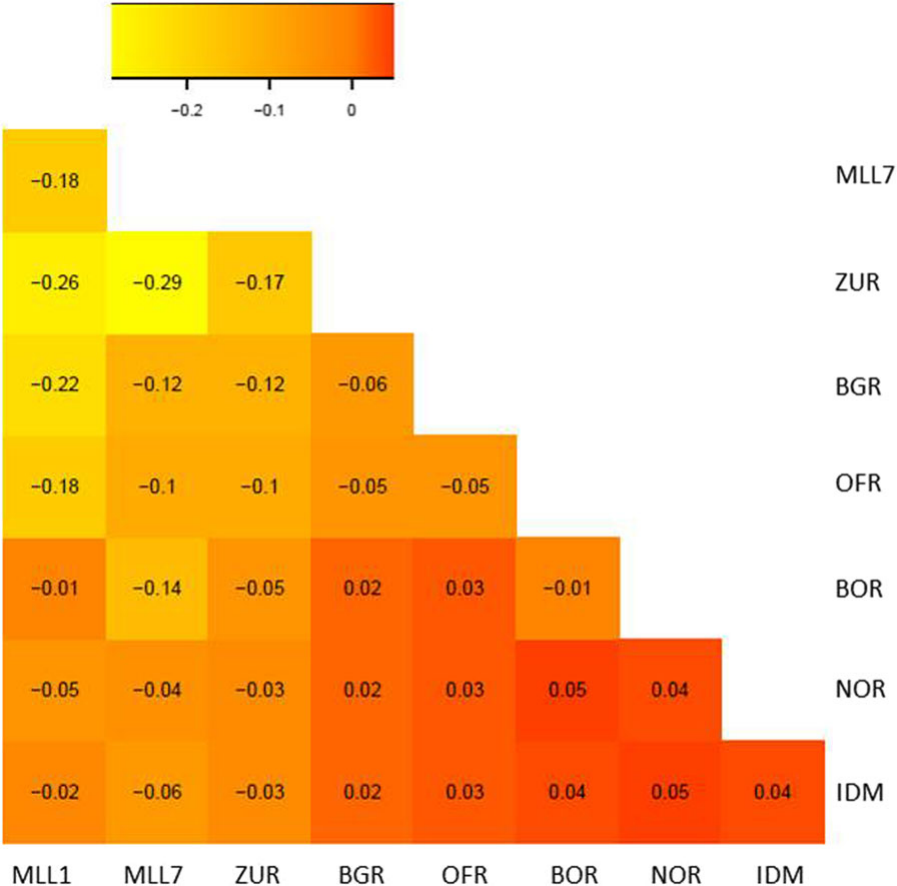
Heat-map illustration of Rousset’s genetic distances between naturally growing populations and multi-locus lineages MLL1 and MLL7, common to scions and rootstocks of grafted old olive trees [33]

Rousset’s â values (Fig. 3) indicated close genetic similarity between trees from populations ZUR and BGR and MLL1 (-0.26 and -0.22, respectively) and between ZUR and MLL7 (-0.29). Considering that trees of population ZUR were assigned to only three MLLs (MLL1, 7 and 269; Table 2), these results are not surprising. Trees from OFR also showed relatively high similarity with MLL1 (-0.18), whereas NOR, BOR and IDM were found to be most divergent (-0.05, -0.01 and -0.02, respectively; Fig. 3). Comparisons with MLL7 showed a similar pattern, except for individuals from BOR which are relatively more similar to MLL7 (-0.14) than to MLL1.

### Population genetic structure

**Fig. 4:**
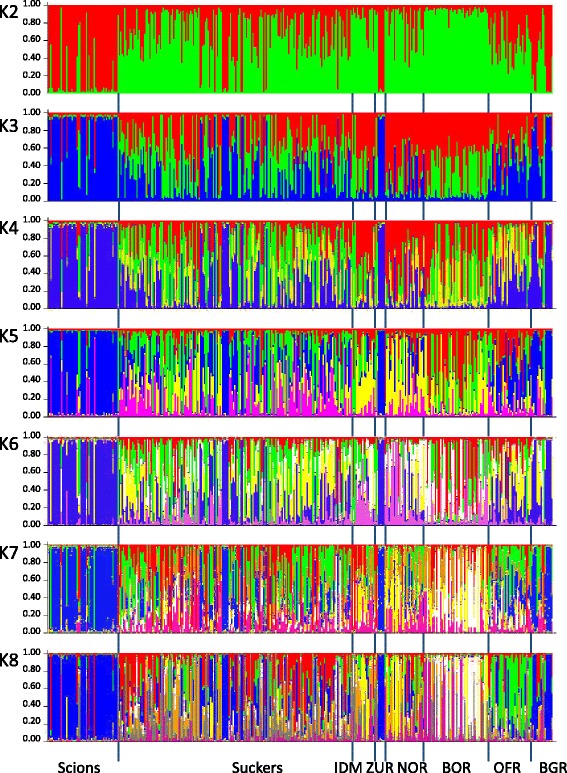
Inferred genetic structure of scions and rootstocks of grafted old olive trees and naturally growing populations of olive trees in the southeast Mediterranean. Bayesian clustering with the admixture model implemented in Structure was used to assign individual MLGs to genetic clusters (K = 3). Individual MLGs within each group are represented by vertical bars and genetic groups are shown in different colors

Results of the Structure analysis are provided for K = 2 to 8 (Fig. 4). A clear peak of ∆K suggested that K = 3 is the optimal number of subgroups (Additional file 5: Figure S2).

Confirming the MLL analysis, MLGs of scions were found to be fairly homogenous at all given Ks. The dominance of the scion cluster in the cultivated olive individuals sampled in BGR was also evident at all Ks, and a similar genetic structure was also found in trees from ZUR (Fig. 4). Naturally growing trees at BGR showed evidence of admixture and resembled the genetic structure found in suckers (K = 3 in Fig. 4,). At K = 3, the other naturally growing populations from the Galilee (IDM) and Carmel (NOR, BOR and OFR) also showed an admixed genetic structure resembling that found among suckers.

## Discussion

The existence of wild olive trees (*Olea europaea* subsp. *europaea* var. *sylvestris*) in Israel was here investigated by using SSR variation in naturally growing populations. Based on a Bayesian analysis of SSR markers, it has recently been suggested that 38 trees sampled outside cultivated groves in Israel are presumably feral [25]. However, although that analysis included supposedly wild ‘*oleaster’* trees that were collected within (Upper and Lower Galilee, Carmel) and outside (Ashkelon, Coastal Plain and Jerusalem) the putative distribution range of var. *sylvestris*, it did not include the most common cultivars in the southeast Mediterranean region, which renders the conclusions rather hypothetical. Here, a genetic comparison between grafted old olive trees (i.e. 288 scions and 281 suckers) and naturally growing olive trees from different populations in Israel provided more comprehensive information on their identity as feral, cultivated or genuinely wild, and furthermore allowed us to obtain evidence for the possible source of rootstocks of grafted old trees.

Trees at BGR showed close genetic similarity to scions of old olive trees, most strongly to MLL1 (Table 2 and Figs. 3 and 4). MLL7, the most common lineage among rootstocks of grafted old olive trees [33] was represented once in this population, and diversity values, estimated as allelic richness and mean number of private alleles per locus, were among the lowest of all populations analyzed (Table 3). Considering the dominance of MLL1 in the supposedly cultivated trees of BGR (Table 2) and the similarity of their genetic structure to scions (Fig. 4), our results confirm our a priori assumption of the existence of an abandoned grove at this site.

Similarly but unexpectedly, most of the trees at ZUR (Galilee), where wild var. *sylvestris* potentially can grow, were very similar to BGR in their genetic composition and genetic structure. A high number of the sampled trees in ZUR belonged to MLL1 (86.9%), two trees were assigned to site specific MLLs, and MLL7 was present in one individual (Table 2). In addition, genetic differentiation between trees from ZUR and MLL1 and MLL7, as measured by Rousset’s value, was the lowest among all pairwise comparisons (Fig. 3), and the genetic diversity values were lowest of all populations investigated (Table 3). Thus, although the ZUR site is best characterized as Mediterranean garrigue (Fig. 1c), and has no resemblance with a grove in terms of tree spacing, traces of former terraces, etc., our results indicate that most trees at this site represent an old abandoned olive grove.

The remaining four populations (IDM, NOR, BOR, OFR) are very different in their genetic composition from BGR and ZUR. Trees from IDM (Galilee) and the three Carmel populations (BOR, NOR and OFR) were found to be similar in terms of relatively high genetic diversity values (Table 3), and genetic differentiation from both BGR and ZUR (abandoned groves) and from MLL1 and MLL7 is high (Fig. 3). However, while the Carmel populations contain large numbers of single occurrence MLLs (≥69% of the total sample size), higher than those found in suckers of cultivated trees (43%; Table 2), 68% of IDM samples belonged to five site-specific MLLs (Table 2), indicating a high frequency of clonal reproduction and/or inbreeding. Indeed, we found indications of inbreeding in IDM (Ho < He, Table 3). This probably can be interpreted as evidence for small effective population size and a high degree of isolation of this population from others. Beyond these peculiarities of IDM, this population and BOR, NOR and OFR fall into two groups. Whereas NOR and IDM do not contain the common scion (MLL1) or rootstock (MLL7) MLLs, both OFR and BOR contain the rootstock MLL7 (Table 2), explaining their similarity to the common rootstock genotype (mean â = -0.10 and -0.14, respectively; Fig. 3), and OFR also contains the scion MLL1. Furthermore, IDM and NOR showed the highest number of private alleles and of alleles that are private to the combination of two populations (Fig. 2 and Additional file 4: Figure S1), and, among these four populations, are most similar to each other (Table 4). The number of private alleles per locus was higher in a combination of suckers, IDM and NOR than in the combination of suckers, BOR and OFR (Additional file 4: Figure S1B). Finally, considering the Structure analysis at K = 3 (Fig. 4), IDM and NOR appear to be more similar to suckers than BOR and OFR in terms of variation of admixed genotypes. In BOR, genotypes with a high proportion of red and green and a low proportion of blue are common, whereas in OFR a relatively high proportion of blue is common. Genotypes are more variably admixed in IDM and NOR. Taking all evidence together, IDM and NOR are most distinct from BGR and ZUR, and BOR and OFR have a somewhat intermediate genetic structure. This in our opinion allows two interpretations:

First, IDM and NOR should be considered wild populations. This interpretation would confirm previous reports that indicated that supposedly wild populations from Carmel and Galilee genetically resemble wild olive populations from Turkey and Syria [17, 19, 21, 26]. As we had demonstrated before [33] that the majority of old olive trees in the southeastern Mediterranean were maintained by grafting (>80%), the similarity in genetic structure between IDM and NOR on the one hand and suckers of old cultivated olive trees on the other hand would imply that scions were grafted on wild growing olive trees (var. *sylvestris*). Similarly, a recent genetic survey of scions and rootstocks of old olive trees in the Iberian Peninsula suggested that old olive trees were grafted on wild growing trees [35]. Based on the distances between grafted trees and their spatial arrangement within the groves, Diez et al. [35] additionally suggested that natural forests were transformed into olive orchards by grafting. In contrast to the situation in Spain, traditional olive groves in the southeastern Mediterranean are found in terraces with equal distances between trees, suggesting that in this region grafting was practiced in the grove itself. This would imply that scions were grafted on saplings that could have been transplanted from outside the grove or germinated in nurseries within it.

Second, the interpretation of the similarity in genetic structure between IDM and NOR on the one hand and suckers of old cultivated olive trees on the other hand can be reversed: such interpretation would imply that the naturally growing trees at IDM and NOR are remnants of old cultivated trees in which the scion-derived trunk died and was replaced by suckers. If this interpretation of IDM and NOR as essentially feral should be correct, populations BOR and OFR may represent an intermediate stage in the transition of orchards into naturally growing populations of feral origin. However, as naturally growing trees at NOR and IDM grow in conditions that are unsuitable for agriculture (c.f. Fig. 1b), it seems more likely to us that they represent wild than abandoned cultivated trees taken over by their suckers.

## Conclusions

The comparison of naturally growing olive tree populations with MLL genotypes of scions and suckers of old cultivated olive trees in Israel allowed us to assess the status of naturally growing populations as abandoned groves, feral or wild var. *sylvestris*. The interpretation of two of six populations analyzed as wild var. *sylvestris* implies that grafting in the past used wild plants as rootstocks. In an area where olive cultivation has a history of several thousand years, it is astonishing to have identified naturally growing olive tree populations which are partly well-differentiated from cultivated plants. Considering the high abundance of cultivated and feral olive trees in the region, conservation of this valuable genetic material is of greatest importance.

## Methods

### The studied populations

Naturally growing olive trees can be found sparsely in Israel in natural habitats surrounding cultivated groves and residential areas. Surveys were conducted in the Carmel and Galilee to locate populations of at least 20 olive trees (*O. europaea* subsp. *europaea*) growing in natural surroundings and resembling the shrubby phenotype of southeastern Mediterranean var. *sylvestris*; identification of plants was based on the Analytical Flora of Israel [36] and Flora Palaestina [37] and done by Dr. Ori Fragman-Sapir (Head Scientist, Jerusalem Botanical Gardens). Naturally growing olive (var. *sylvestris*) is not included in the Red List of the Israeli Flora [38]. Nevertheless, sampling in natural reserves was coordinated and approved by the Israel Nature and National Parks Protection Authority (license no. 2014/40360).

Five sites were selected (Table 1; Fig. 1a) within the species’ hypothetical natural distribution range in the region (Galilee and Carmel Mts.), and one population was sampled in the Judean Mts. outside the hypothetical natural distribution range of wild olive [9]. At all sites, olive trees were growing at uneven distances which is untypical for cultivated groves. Two populations were sampled in the Galilee, three in the Carmel Mountain range and one in the Judean Mts. (Table 1; Fig. 1a). Number of samples collected is related to population size. Where possible, trees were sampled randomly along widely spaced transects through the collecting areas in order to represent their genetic diversity; this resulted in a sample of altogether 205 trees. As the Carmel Mountain range is considered the southern limit of the distribution range of wild olive trees in Israel [9], the populations in the Galilee and the Carmel regions potentially may represent wild var. *sylvestris*.

At Idmit (Galilee; IDM), the sampled population grows remotely from contemporary olive groves (Additional file 6: Figure S3) as a dense stand on the edge of a cliff with a southwest slope facing the Mediterranean Sea (335 m above sea level; a.s.l). Trees at IDM face strong herbivore pressure, mainly by rock-hyrax (*Procavia capensis*), and grow in patches as small shrubs (Fig. 1b), different from the trees at the other sites. At IDM, 25 trees were sampled. At the second site, sampled in the western Galilee (ZUR; Zurit, 295 m a.s.l.), closer to an olive cultivation area (~3 km), trees were observed in scattered patches across a large area of about 50 hectares (Fig. 1c). In order to represent the genetic diversity of this population, 23 samples were collected from across the entire area. In the Carmel region, 128 trees were sampled in three locations: (1) on south and north facing slopes of the western part of Nachal Oren (NOR, 140 m a.s.l., 35 trees), a natural habitat that is not suitable for agriculture and has long been used as a model site for biodiversity and speciation studies [39]; (2) at a higher point of the Carmel Mountain in the vicinity of Beit Oren (BOR, 385 m a.s.l., 54 trees); (3) in the southern part of the Carmel range (Ofer), trees were sampled on north and south facing slopes of the hill (OFR; 170 m a.s.l., 39 trees). At the last site, trees are distributed irregularly, untypical for olive groves, and tree appearance is different from nearby cultivated trees found at the edge of a rural residential area. Hypothesizing that the OFR population might be feral, we included two cultivated trees from the residential area in our analysis; OFR is also relatively close to an old olive grove (32° 37′ 48.00″N, 35° 0′ 0.00″E) which had been sampled in our previous study [33] (Additional file 6: Figure S3). At Bar Giora (BGR; Judean Mts.; 450 m a.s.l.), trees were sampled in a natural reserve on northeast facing slope terraces in the remains of an abandoned orchard and thus were assumed to represent a population of non-wild olives. Of the total of 29 trees sampled here, 11 resembled cultivated trees; however, since unambiguous distinction of cultivated from naturally growing trees was not possible, the 29 trees were treated as one group. The Galilee (IDM, ZUR) and Carmel (NOR, OFR) areas can be characterized as garrigue (Fig. 1); olive trees in BOR and BGR grow in Mediterranean dense forest (maquis).

### Genetic analysis

DNA was extracted from leaf samples using the Invisorb Plant Mini Kit (Invitek) following the manufacturer’s protocol. Simple Sequence Repeat (SSR) markers used in olive trees [40–48] had previously been screened [33] resulting in the use of 15 markers with PCR conditions previously described (Additional file 1: Table S1) [33]. SSR products were separated at the Center of Genomic Technologies (The Hebrew University of Jerusalem) on an ABI automated sequencer (Applied Biosystems) as a multiplex of several loci labeled with three different fluorescent dyes (6-FAM, NED and HEX; Applied Biosystems). Electropherograms were scored manually using Genmarker 1.75 (SoftGenetics, State College, Pennsylvania, USA).

Analysis of multi-locus genotypes (MLGs) and grouping of MLGs into multi-locus lineages (MLLs) was done using Genotype 1.2 [49]. To estimate diversities, one sample of the most common MLG of each MLL from each population was used. Genetic diversity values calculated included observed (Ho) and unbiased expected (uHe) heterozygosity using GenAlEx v6.5 [50, 51]. To better account for differing sample sizes, allelic and private allelic richness was calculated with a rarefaction approach using the Allelic Diversity Analyzer ADZE software [34]. ADZE was also used to calculate the number of alleles private to combinations of populations and scions and suckers of old cultivated trees. The diversity values obtained were compared with data from 281 suckers collected at the base of tree trunks and 288 scions of cultivated old olive trees [33] using the same 15 SSR loci as used here. The old cultivated olive trees were sampled in 32 groves in the southeastern Mediterranean; the location of the groves is listed in Barazani et al. [33]. The ZUR population included only three different MLLs and was excluded from analyses based on MLLs. In addition, Fst values were used to estimate genetic distances among populations, using GenAlEx v6.5. Rousset’s genetic distances (â) [52] were estimated using Spagedi 1.4c [53] to compare genetic distances among individuals within and between populations as well as genetic distances to MLL1 and MLL7, the two most commonly cultivated clones in the region [33].

Structure *V*.2.3.4 [54] was used for Bayesian clustering with the admixture model to assign each MLG from each of the studied naturally growing populations and from old cultivated trees to K clusters. According to the recommendation by Pritchard et al. [55], 10 independent runs for given Ks (2 to 8) were performed with a burn-in length of 10,000, followed by 20,000 repetitions. The log likelihoods for a given K were used to choose the best given K based on an ad hoc quantity of ∆K [56].

## Additional files

Additional file 1: Table S1. SSR markers used, their expected size range, repeated motives and number of alleles found in naturally growing olive populations. Raw microsatellite data is available and enclosed as Additional file 2: Table S2. (PDF 188 kb)
Additional file 2: Table S2. Raw microsatellite data. The fragment sizes (in base pairs) of the two alleles per individual for each locus are given as a and b (0 represents missing data). (XLSX 38 kb)
Additional file 3: Table S3. Number of olive trees assigned to different multi-locus lineages (MLLs). (XLSX 18 kb)
Additional file 4: Figure S1. Number of private alleles per locus in combinations of populations. A to D present values for the combination of two to five populations (treating scions and suckers of old olive trees as populations). (PDF 217 kb)
Additional file 5: Figure S2. ∆K values for the different Ks were calculated according to Evanno et al. [56], showing that K = 3 is the optimal K for the Structure analysis. (PDF 69 kb)
Additional file 6: Figure S3. Location of populations of naturally growing olives analyzed in this study and of groves of cultivated old olive trees sampled in our previous study (Barazani et al. [33]). (PDF 79 kb)

## Abbreviations

BGR: Naturally growing olive population at Bar Giora
BOR: Naturally growing olive population at Beit Oren
IDM: Naturally growing olive population at Idmit
MLG: Multi-locus genotype
MLL: Multi-locus lineage
NOR: Naturally growing olive population at Nachal Oren
OFR: Naturally growing olive population at Ofer
SSR: Simple sequence repeat
ZUR: Naturally growing olive population at Zurit
