# Supplementary material for: Genetic variation of naturally growing olive trees in Israel: from abandoned groves to feral and wild?
Source: BMC Plant Biol. 2016 Dec 13;16:261. doi: 10.1186/s12870-016-0947-5 (PMC5154132; doi:10.1186/s12870-016-0947-5)
Supplement: Additional file 1: Table S1. — SSR markers used, their expected size range, repeated motives and number of alleles found in naturally growing olive populations. Raw microsatellite data is available and enclosed as Additional file 2: Table S2. (PDF 188 kb) [file 12870_2016_947_MOESM1_ESM.pdf]

Table S1. SSR markers used, their expected size range, repeated motives and number of alleles found in naturally growing olive populations. Raw microsatellite data is available in Table S2 (enclosed as an Excel file).

SSR Marker	Expected range	Repeat motif	Reference	# Alleles
IAS-oli26	168-210	$(GA)_{14}$	Diaz et al. 2006	10
UDO99-011	115	$(CT)_{7}(CA)_{10}(CT)_{2}(CA)_{2}CT(CA)_{2}CT(CA)_{9}$	Cipriani et al. 2002	8
UDO99-024	188	$(CA)_{11}(TA)_{2}(CA)_{4}$	Cipriani et al. 2002	12
UDO99-025	158	$(AC)_{16}(AT)_{5}$	Cipriani et al. 2002	16
UDO99-043	174	$(GT)_{12}$	Cipriani et al. 2002	32
ssrOeUA-DCA1	230 (204–230)	$(GA)_{22}$	Sefc et al. 2000	8
ssrOeUA-DCA3	250 (228–250)	$(GA)_{19}$	Sefc et al. 2000	30
ssrOeUA-DCA9	191 (161–205)	$(GA)_{23}$	Sefc et al. 2000	24
ssrOeUA-DCA11	179 (125–161)	$(GA)_{26}(GGGA)_{4}$	Sefc et al. 2000	23
sssrOeUA-DCA16	178 (120–178)	$(GT)_{13}(GA)_{29}$	Sefc et al. 2000	29
ssrOeUA-DCA18	178 (168–184)	$(CA)_{4}CT(CA)_{3}(GA)_{19}$	Sefc et al. 2000	13
PA (ATT)2	115–136	$(TAA)_{6}$	Saumitou-Laprade et al. 2000	4
EMO03	205-215	$(CA)_{7}$	De la Rosa et al. 2002	12
EMO90	184 (180–197)	$(CA)_{10}$	De la Rosa et al. 2002	13
GAPU59	227	$(CT)_{9}$	Carriero et al. 2002	13
