# Supplementary material for: Genetic variation of naturally growing olive trees in Israel: from abandoned groves to feral and wild?
Source: BMC Plant Biol. 2016 Dec 13;16:261. doi: 10.1186/s12870-016-0947-5 (PMC5154132; doi:10.1186/s12870-016-0947-5)
Supplement: Additional file 4: Figure S1. — Number of private alleles per locus in combinations of populations. A to D present values for the combination of two to five populations (treating scions and suckers of old olive trees as populations). (PDF 217 kb) [file 12870_2016_947_MOESM4_ESM.pdf]

Figure S1. Number of private alleles per locus in combinations of populations. A to D present values for the combination of two to five populations (treating scions and suckers of old olive trees as populations).

A

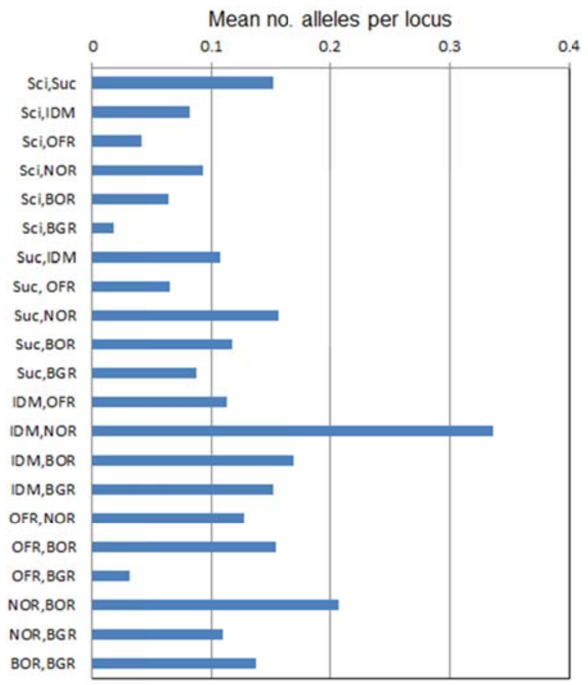

B

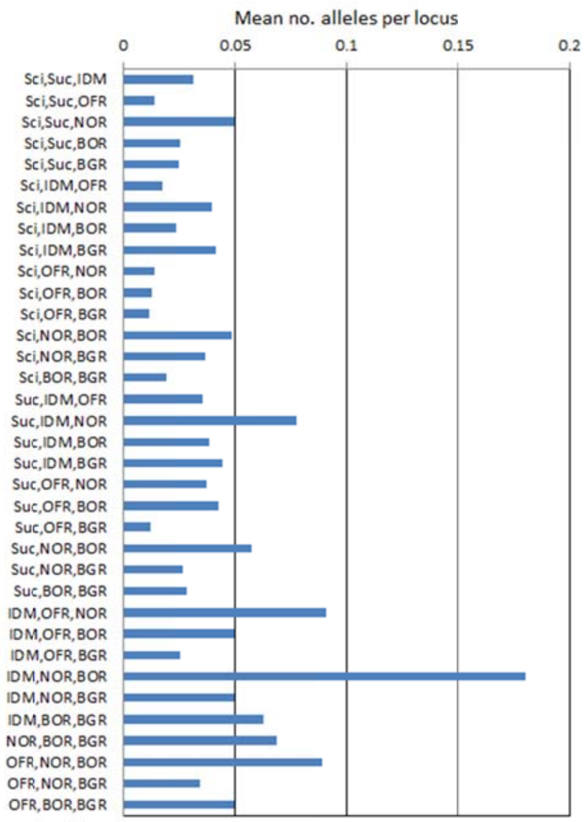

C

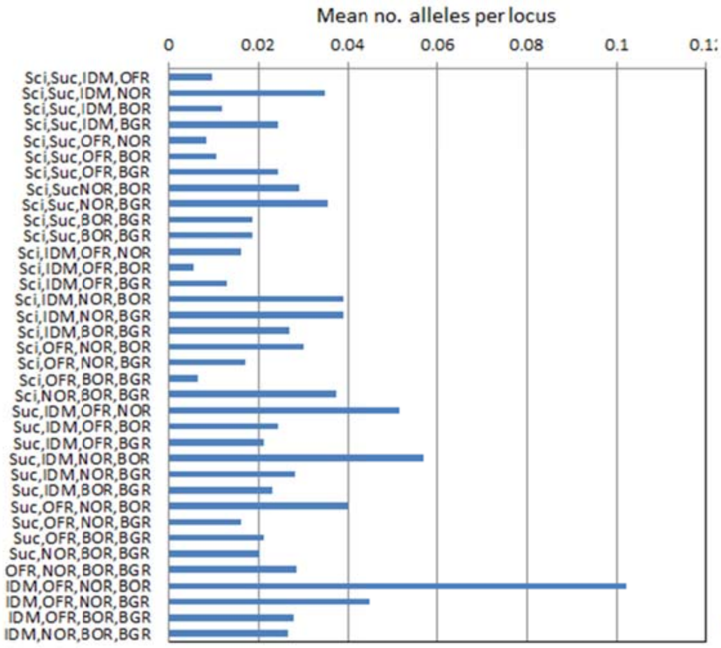
Mean no. alleles per locus
0
0.02
0.04
0.06
0.08
0.1
Sci,Suc,IDM,OFR
Sci,Suc,IDM,NOR
Sci,Suc,IDM,BOR
Sci,Suc,IDM,BGR
Sci,Suc,OFR,NOR
Sci,Suc,OFR,BOR
Sci,Suc,OFR,BGR
Sci,SucNOR,BOR
Sci,Suc,NOR,BGR
Sci,Suc,BOR,BGR
Sci,Suc,BOR,BGR
Sci,IDM,OFR,NOR
Sci,IDM,OFR,BOR
Sci,IDM,OFR,BGR
Sci,IDM,NOR,BOR
Sci,IDM,NOR,BGR
Sci,IDM,BOR,BGR
Sci,OFR,NOR,BOR
Sci,OFR,NOR,BGR
Sci,OFR,BOR,BGR
Sci,NOR,BOR,BGR
Suc,IDM,OFR,NOR
Suc,IDM,OFR,BOR
Suc,IDM,OFR,BGR
Suc,IDM,NOR,BOR
Suc,IDM,NOR,BGR
Suc,IDM,BOR,BGR
Suc,OFR,NOR,BOR
Suc,OFR,NOR,BGR
Suc,OFR,BOR,BGR
Suc,NOR,BOR,BGR
OFR,NOR,BOR,BGR
IDM,OFR,NOR,BOR
IDM,OFR,NOR,BGR
IDM,OFR,BOR,BGR
IDM,NOR,BOR,BGR

D

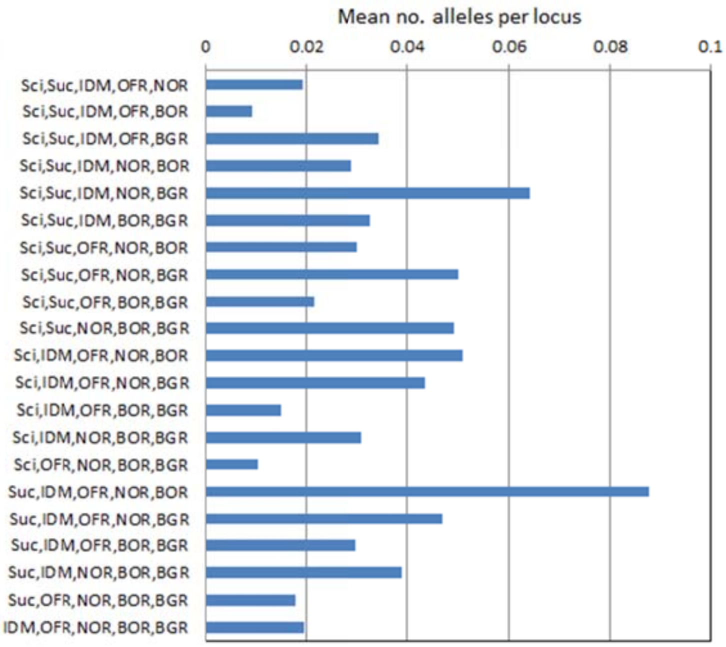
Mean no. alleles per locus
0
0.02
0.04
0.06
0.08
0.1
Sci,Suc,IDM,OFR,NOR
Sci,Suc,IDM,OFR,BOR
Sci,Suc,IDM,OFR,BGR
Sci,Suc,IDM,NOR,BOR
Sci,Suc,IDM,NOR,BGR
Sci,Suc,IDM,BOR,BGR
Sci,Suc,OFR,NOR,BOR
Sci,Suc,OFR,NOR,BGR
Sci,Suc,OFR,BOR,BGR
Sci,Suc,NOR,BOR,BGR
Sci,IDM,OFR,NOR,BOR
Sci,IDM,OFR,NOR,BGR
Sci,IDM,OFR,BOR,BGR
Sci,IDM,NOR,BOR,BGR
Sci,OFR,NOR,BOR,BGR
Suc,IDM,OFR,NOR,BOR
Suc,IDM,OFR,NOR,BGR
Suc,IDM,OFR,BOR,BGR
Suc,IDM,NOR,BOR,BGR
Suc,OFR,NOR,BOR,BGR
IDM,OFR,NOR,BOR,BGR
