# Supplementary material for: Genetic variation of naturally growing olive trees in Israel: from abandoned groves to feral and wild?
Source: BMC Plant Biol. 2016 Dec 13;16:261. doi: 10.1186/s12870-016-0947-5 (PMC5154132; doi:10.1186/s12870-016-0947-5)

Figure S2. ΔK values for the different Ks were calculated according to Evanno et al. (2005), showing that K=3 is the optimal K for the Structure analysis.

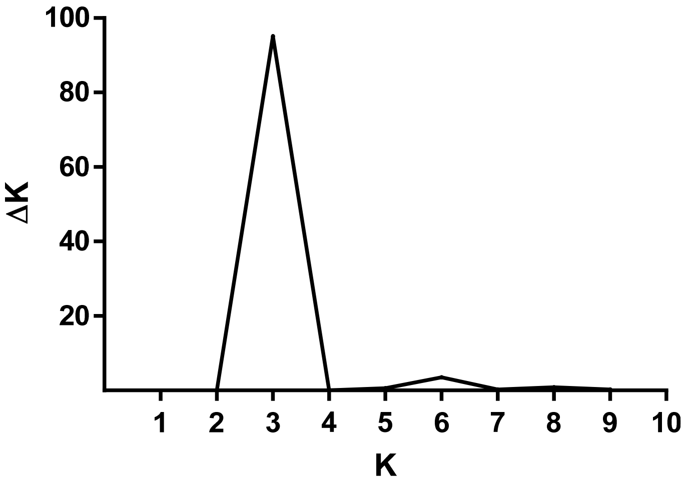

Supplement: Additional file 5: Figure S2. — ∆K values for the different Ks were calculated according to Evanno et al. [56], showing that K = 3 is the optimal K for the Structure analysis. (PDF 69 kb) [file 12870_2016_947_MOESM5_ESM.pdf]
