# Supplementary material for: Genetic variation of naturally growing olive trees in Israel: from abandoned groves to feral and wild?
Source: BMC Plant Biol. 2016 Dec 13;16:261. doi: 10.1186/s12870-016-0947-5 (PMC5154132; doi:10.1186/s12870-016-0947-5)

Figure S3. Location of populations of naturally growing olives analysed in this study and of groves of cultivated old olive trees sampled in our previous study (Barazani et al. 2014 [33])

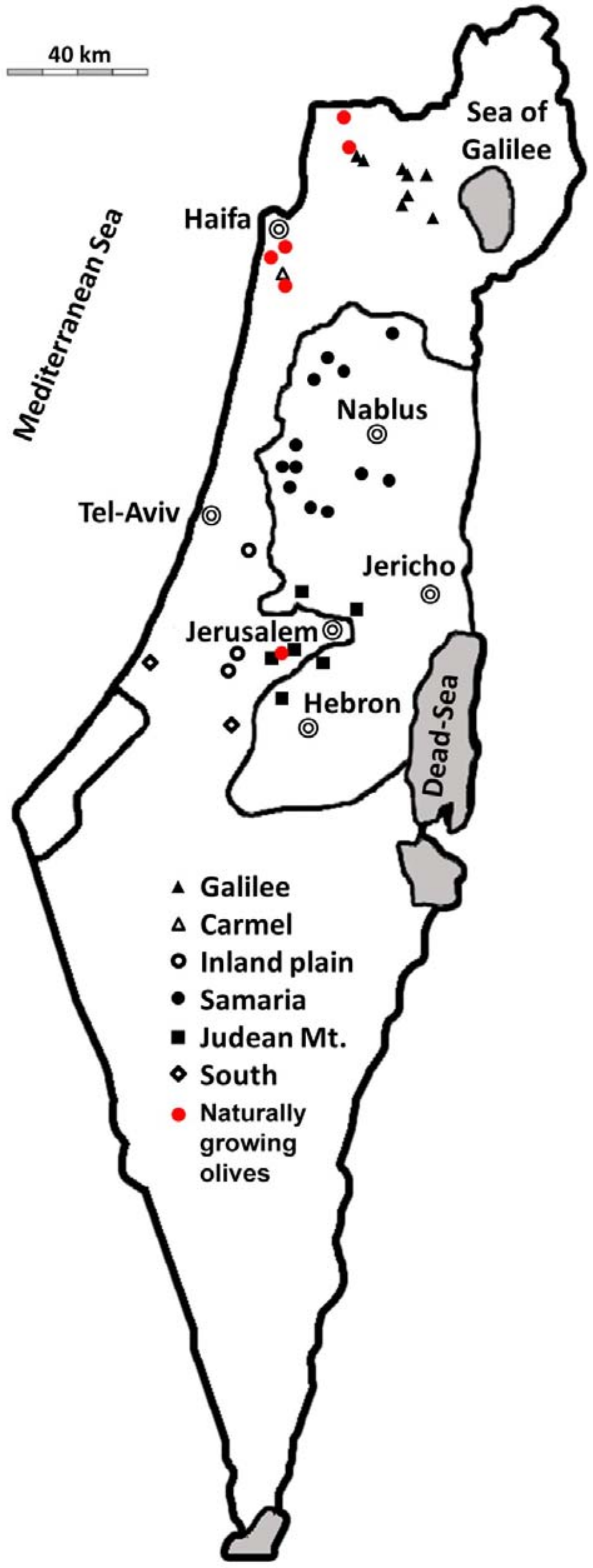

Supplement: Additional file 6: Figure S3. — Location of populations of naturally growing olives analyzed in this study and of groves of cultivated old olive trees sampled in our previous study (Barazani et al. [33]). (PDF 79 kb) [file 12870_2016_947_MOESM6_ESM.pdf]
